# Simultaneous deletion of *3′ETV6* and *5′EWSR1* genes in blastic plasmacytoid dendritic cell neoplasm: case report and literature review

**DOI:** 10.1186/s13039-016-0232-1

**Published:** 2016-02-27

**Authors:** Zhenya Tang, Guilin Tang, Sa A. Wang, Xinyan Lu, Ken H. Young, Carlos E. Bueso-Ramos, Yesid Alvarado, L. Jeffrey Medeiros, Joseph D. Khoury

**Affiliations:** Department of Hematopathology, The University of Texas MD Anderson Cancer Center, 1515 Holcombe Boulevard, Houston, TX 77030 USA; Department of Leukemia, The University of Texas MD Anderson Cancer Center, 1515 Holcombe Boulevard, Houston, TX 77030 USA

**Keywords:** Blastic plasmacytoid dendritic cell neoplasm (BPDCN), Karyotype, Chromosomal abnormality, 12p-, *ETV6*, *CNKN1B*, *EWSR1*

## Abstract

**Background:**

Blastic plasmacytoid dendritic cell neoplasm (BPDCN) is a rare hematologic malignancy. Based on literature reports of limited cases, over 50 % of BPDCN have chromosomal abnormalities, but no single chromosomal change has been identified as diagnostic of this entity.

**Case presentation:**

In this report, we present a case of BPDCN with complicated chromosomal abnormalities involving chromosomes 12 and 22 and resulting in a simultaneous partial deletion of *ETV6* and *EWSR1*. Notably, these aberrations were identified in bone marrow myeloid precursors in the absence of bone marrow involvement by BPDCN.

**Conclusion:**

Analysis of 46 BPDCN cases with abnormal karyotypes (45 from literature reports plus this case) showed that 12p- is one of the most common structural aberrations in BPDCN. The *ETV6* and *CDKN1B* on 12p deserve further investigations as potential markers of BPDCN.

## Background

Blastic plasmacytoid dendritic cell neoplasm (BPDCN) is a rare, aggressive myeloid neoplasm derived from plasmacytoid dendritic cells [1]. This disease has been previously described in the past using terms such as “acute agranular CD4+ natural killer (NK) cell leukemia” [2], “blastic NK cell lymphoma” [3] and “agranular CD4 + CD56+ hematodermic neoplasm/tumor” [4, 5]. BPDCN may involve multiple sites, commonly skin, bone marrow (BM), peripheral blood (PB) and lymph nodes (LN). Based on literature reports of limited cases, over 50 % of BPDCN have chromosomal abnormalities, but no single chromosomal change has been shown to be diagnostic of this entity. The common chromosomal aberrations in BPDCN reported previously include abnormalities involving chromosomes 5q (72 %), 12p (64 %), 13q (64 %), 6q (50 %), 15q (43 %), and 9 (usually monosomy 9, 28 %) [4, 6]. Aberrations of 12p are among the most common findings in BPDCN.

In this report, we present a case of BPDCN with complicated chromosomal abnormalities involving chromosomes 12 and 22 and resulting in simultaneous partial deletion of *ETV6* and *EWSR1*. These findings were identified in the BM of a patient with BPDCN in the absence of morphologic, immunohistochemical, or flow cytometry evidence of BPDCN. We also conduct a literature review of conventional cytogenetic findings in BPDCN.

## Case presentation

The patient is a previously healthy 44-year-old man who presented with a painless enlarging mass in his left groin. He was observed initially for three months and eventually was referred for an excisional lymph node biopsy. Histologic examination showed a high-grade malignant neoplasm that was diagnosed as BPDCN. He was then referred to our institution. BM evaluation included a trephine biopsy and aspiration. There was no evidence of BPDCN in BM by morphology or immunohistochemistry. Flow cytometry was also negative for BPDCN in BM. However, conventional cytogenetic analysis performed on the BM aspirate sample showed karyotypic aberrations involving chromosomes 12 and 22, which were further characterized by fluorescence in situ hybridization (FISH) analysis (see details below). The patient was treated with a hyper-CVAD-Bortezomib regimen (hyperfractioned cyclophosphamide, vincristine, doxorubicin, dexamethasone alternating with high dose of methotrexate and cytarabine, plus bortezomib) regimen. He also received prophylactic intrathecal chemotherapy with methotrexate for 3 cycles and achieved a complete remission.

## Methods and results

### Conventional chromosomal analysis

Conventional chromosomal analysis (karyotyping) was performed on G-banded metaphase cells prepared from unstimulated 24-h and 48-h bone marrow cultures as described previously [7]. Twenty metaphases (10 from each culture) were analyzed. The chromosomal abnormalities were reported according to the International System for Human Cytogenetic Nomenclature 2013 (*ISCN2013*) guidelines [8]. Out of 20 metaphases analyzed, 10 exhibited structural abnormalities involving chromosomes 12 and 22.

### Fluorescence in situ Hybridization (FISH) analysis

The following FISH probes were applied in this study: Vysis *ETV6* Break Apart (BAP) FISH probe and Vysis *EWSR1* BAP FISH probe (Abbott Molecular, Des Plaines, IL) were used for interphase, metaphase and tissue FISH tests. The Vysis *MYC* BAP FISH probe (Abbott Molecular, Des Plaines, IL) was used for both interphase and tissue FISH tests. The Vysis LSI *BCR/ABL* ES Dual Color Fusion probe (Abbott Molecular, Des Plaines, IL) was used for interphase FISH tests, whereas the Aquarius Whole Chromosome Painting (WCP) probes for chromosomes 12 and 22 (Cytocell, Tarrytown, NY) were used for metaphase FISH only. All probes were thoroughly validated in accordance with the American College of Medical Genetics and Genomics (ACMGG) guidelines.

**Fig. 1:**
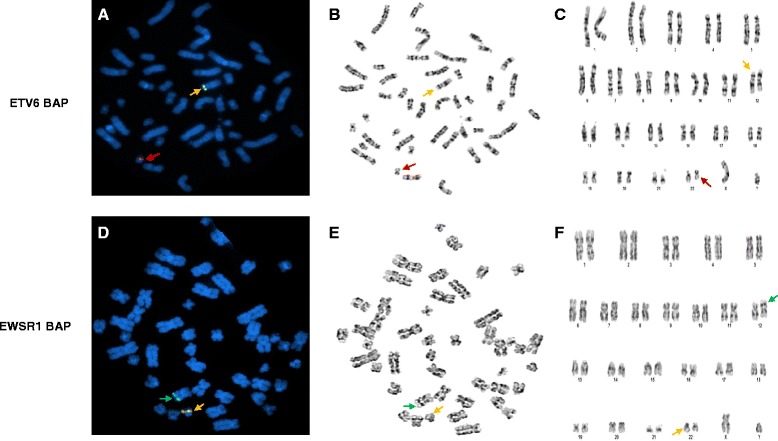
Mapping back to G-banded Metaphases with *ETV6* BAP and *EWSR1* BAP respectively. *ETV* BAP FISH (**a**-**c**): **a**. Metaphase FISH exhibiting an intact *ETV6* (yellow signal) on a normal chromosome 12 and *5′ETV6* (red signal) on an abnormal chromosome 22; **b**. Metaphase; **c**. Karyotype. *EWSR1* BAP FISH (**d**-**f**): **d**. Metaphase FISH exhibiting an intact *EWSR1* (yellow signal) on a normal chromosome 22 and *3′EWSR1* (green signal) on an abnormal chromosome 12; **e**. Metaphase; **f**. Karyotype

Interphase FISH performed on unstimulated cultured cells from BM sample using the *ETV6* BAP probe showed that about 25 % of cells had a one-red-one-fusion (1R1F) signal pattern, indicating an *ETV6* gene rearrangement with a partial deletion of the *3′ETV6* (green signal). A separate interphase FISH analysis using the *EWSR1* BAP probe showed that almost the same percentage of cells had a one-green-one-fusion (1G1F) signal pattern, demonstrating an *EWSR1* gene rearrangement with a partial deletion of the *5′EWSR1* (red signal). Mapping back to previously G-banded and karyotyped metaphases showed that the existing *5′ETV6* (red signal) is located at the long arm of the abnormal chromosome 22, whereas the existing *3′EWSR1* (green signal) is located at the short arm of the abnormal chromosome 12 (Fig. 1). Whole chromosome painting (WCP) further confirmed the origins as abnormal chromosomes 12 and 22 (images not shown). Therefore, the results of conventional cytogenetic analysis and FISH analysis suggest that a translocation between 12p and 22q occurred. This was likely followed by a pericentric inversion of the abnormal chromosome 12 resulting in a partial deletion of both *3′ETV6* and *5′EWSR1*. These two abnormal chromosomes are described as der(12)t(12;22)(p13;q12)del(22)(q12q12)inv(12)(p13q24.1) and der(22)t(12;22)del(12)(p13p13).

**Fig. 2:**
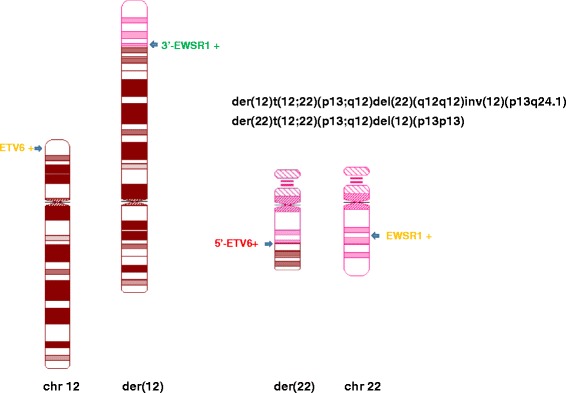
Karyograms of normal chromosomes 12 and 22, abnormal chromosomes 12 (der(12)) and 22 (der922)) drawn by using CyDAS program [9] with indication of sites and colors of *ETV6* BAP and *EWSR1* BAP of FISH tests in this study

Due to the complexity of these chromosomal aberrations and a low resolution of the available karyogram, the derivative chromosomes were drawn by using the online CyDAS software [9], and the corresponding FISH signals were labeled (Fig. 2). Neither *MYC* gene rearrangement nor *BCR/ABL* fusion were detected in the BM specimen.

**Fig. 3:**
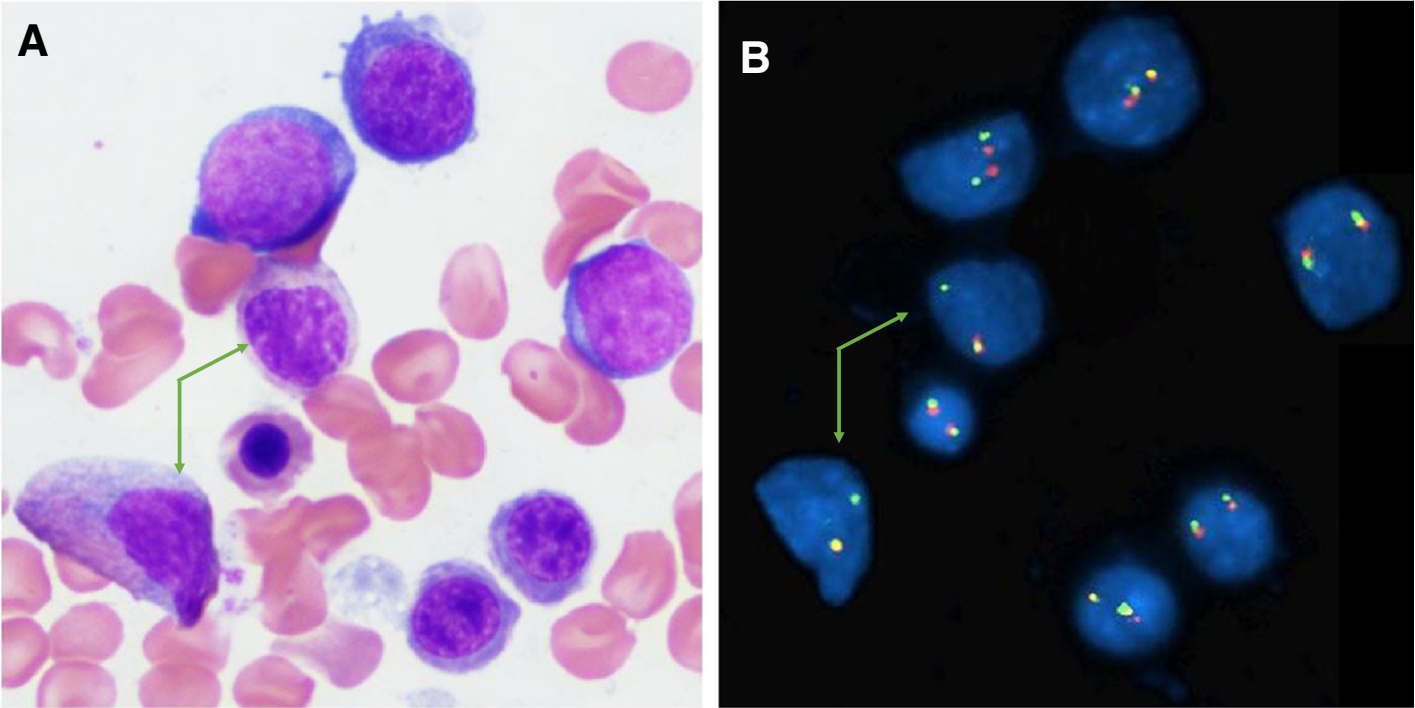
Combined morphology and FISH analysis on the same bone marrow slide to further characterize the type(s) of cells carrying the chromosomal aberrations described above. **a** H.E. staining image (100×) showing all cells morphologically normal; **b** FISH test using *ETV6* BAP probe showing that in the same field as the H.E. staining image, majority of the cells have exhibited two-fusion/yellow signals pattern, except two maturing myelocytes (pointed with green arrows in both **a** and **b**) showing one-fusion/yellow, one-green signals pattern, indicating deletion of one copy of the *3′ETV6* (red signal)

Interphase FISH performed on the formalin-fixed, paraffin-embedded LN sample showed that 90 % of cells had the same *EVT6* and *EWSR1* signal patterns as detected in the BM (images not shown). Therefore, the same chromosomal aberrations have been ​confirmed on FFPE tissues of the LN biopsy as well. We combined morphologic and FISH analysis as described previously [10, 11] to further characterize the type of BM cells carrying the chromosomal aberrations described above. As shown in Fig. 3, all cells in the BM specimen were morphologically and immunopheno typically normal (Fig. 3a, H.E. staining image), but FISH tests using *ETV6* BAP probe on the same slide detected a positive signal pattern for *ETV6* rearrangement and partial deletion of *3′ETV6* (Fig. 3b, FISH test image).

### Morphologic and flow cytometry immunophenotypic analyses

Morphological examination of hematoxylin-eosin-stained histologic sections of BM biopsy specimen and Romanowsky stained PB and/or BM aspirate smears did not show any morphologic evidence of disease. Cell markers including CD2, CD4, CD5, CD7, CD13, CD14, CD15, CD19, CD22, CD33, CD34, CD36, CD38, CD45, CD56, CD64, CD117, CD123, HLA-DR (Becton-Dickinson Biosciences, San Jose, CA) were assessed by flow cytometry immunophenotyping [6, 10, 11]. No evidence of a CD123 positive, CD4 positive cell population was detected.

### Molecular testing

The BM sample was screened for somatic mutations using a clinically validated next-generation sequencing (NGS)-based 28-gene assay [6]. The genes in this panel included: *ABL1*, *ASXL1*, *BRAF*, *DNMT3A*, *EGFR*, *EZH2*, *FLT3*, *GATA1*, *GATA2*, *HRAS*, *IDH1*, *IDH2*, *IKZF2*, *JAK2*, *KIT*, *KRAS*, *MDM2*, *MLL*, *MPL*, *MYD88*, *NOTCH1*, *NPM1*, *NRAS*, *PTPN11*, *RUNX1*, *TET2*, *TP53*, *and WT1*. No mutations in any of the genes assessed were detected in the BM sample.

## Discussion

We report the first case of BPDCN that carried a translocation between chromosomes 12 and 22, followed by a subsequent pericentric inversion of the abnormal chromosome 12, and that resulted in a simultaneous partial deletion of *3′ETV6* and *5′EWSR1*. Based on separate locations of the remaining *5′ETV6* and *3′EWSR1* (Fig. 2), there is unlikely an *ETV6/EWSR1* fusion gene present in this case. Importantly, these aberrations were detected in BPDCN cells in the LN as well as in hematopoietic precursors in a BM samples that had no evidence of involvement by BPDCN.

The cytogenetic characterization of BPDCN is not well established, mostly due to the rarity of this disease and its relatively recent recognition and diagnostic characterization. In one of the largest cohorts to date, Leroux et al. [4] reported that 14 of 21 cases of CD4 + CD56+ DC2 acute leukemia/BPDCN had an abnormal karyotype, which was further characterized using interphase FISH, metaphase FISH, whole chromosome painting (WCP) and spectral karyotyping (SKY). These analyses delineated six major chromosomal targets for this disease, including 5q (72 %), 12p (64 %), 13q (64 %), 6q (50 %), 15q (43 %) and 9 (28 %). Additional smaller studies and reports showed similar findings.

In Table 1, we have summarized a total of 46 BPDCN cases with abnormal karyotypes, 45 from previous literature reports [4, 6, 12–28] plus the case presented in this report. A minor modification has been made to some of the cases from previous literature reports in order to follow the ISCN 2013 nomenclature guidelines [8] as well as to integrate all findings derived by means other than conventional analysis (e.g., FISH and SKY) into the description of an abnormal karyotype. A similar number of BPDCN cases with possible chromosomal abnormalities are not included in this table, mainly due to a lack of complete karyotype description in these literature [29–37]. In addition, our analysis has focused on cytogenetic alterations and does not include mutations in genes such as TET2 that have been shown to be present in a sizeable subset of BPDCN [6, 38]. The mutational landscape of BPDCN is beyond the scope of this review.

**Table 1:** A summary of abnormal karyotypes in BPDCN cases reported in literatures

Case#	Abnormal Karyotypes^a^	Authors
1	44,X,-Y,der(1)t(Y;1)(q12;q?21),der(3)t(1;3)(p11;q?),del(5)(?q33q35),der(6)t(1;6)(q22;q?)t(1;8)(q?;q?),der(12)t(1;12)(?;p11),r(13)[7]/46,XY[8]	Leroux et al. [4]
2	46,XX,del(5)(q13q33)[2]/42,idem,der(1)t(1;8)(?q43;?),dup(2)(?q35q37),-9,der(12)(?),-13,-15,der(20;21)(?p11;?q22),der(21;21)(q21;q11)ins(21;12)(q11;?)[20]/46,XX[3]	Leroux et al. [4]
3	84 ~ 87,XX,del(X)(q24),del(X)(q24),add(2)(q3?),der(2)(?),+del(4)(q23),+del(5)(q14),+del(5)(q14q23),add(6)(q?),-9,-11,del(12)(p12),-13,-13,-14,-15,-15,-17,-17,-18,+r(?),+1 ~ 3mar[cp11]/46,XX[4]	Leroux et al. [4]
4	45,XY,del(5)(q3?1q3?5),der(7)t(7;12)(?p11.2;q11),-12[3]/46,XY[10]	Leroux et al. [4]
5	45,XY,+5,der(5)t(5;11)(p10;?),der(5)t(5;11)(p10;?),add(6)(q?22),del(11)(q14q23),-13,-15[4]/46,XY[2]	Leroux et al. [4]
6	44,XX,del(3)(p21),-5,-12,-13,add(17)(p11),-18,-19,+3mar[27]/46,XX[2]	Leroux et al. [4]
7	48,XY,+6,add(6)(q10),add(6)(q10),add(9)(p24),der(12)t(1;12)(q22;p13),+21[2]/46,XY[13]	Leroux et al. [4]
8	43,XY,t(3;6)(p25;q2?3),der(7)t(7;19)(p21;q10),-9,der(12)t(5;12)(?;p11),-13,-19[10]	Leroux et al. [4]
9	46,XY,del(2)(p2?1),add(8)(q2?4),del(13)(q1?3q2?1)[31]/47,idem,+mar[10]	Leroux et al. [4]
10	46,XX,del(12)(p12p13)[9]/46,XX[2]	Leroux et al. [4]
11	45,XX,del(5)(q13q34),?inv(11)(p11q21),der(15;18)(q10;q10)[15]/44,idem,-22,dmin[2]/88,idemx2,del(6)(q16)[2]/46,XX[1]	Leroux et al. [4]
12	42,X,-Y,der(2)t(2;5)(p?;?)t(2;6)(q?14;q11),del(5)(p13),der(5)t(5;13)(q21;q?),der(6)t(2;6),t(6;18)(q2?2;q22),der(11),-13,der(13)t(13;21)(q10;q10),-14,der(14)t(Y;14)(q11;p11),r(15),der(19)t(3;19)(p21;q13),-21,i(22)(q10)[18]/46,XY[3]	Leroux et al. [4]
13	44,XY,-9,-13[8]/88,idemx2[18]/46,XY[5]	Leroux et al. [4]
14	49,XY,+6,t(6;8)(p21;q24),+r(12),+20[6]/49,idem,inv(15)(q1?4q2?3),t(16;16)(q?;q?)[6]/49,idem,t(3;5)(q?21;q?31)[5]	Leroux et al. [4]
15	46,XX,del(5)(q13q33)[2]/42,idem,-9,-12,-13,-15,-21,+3mar[20]/46,XY[2]	Petrella et al. [12]
16	43,XX,der(1)t(1;15)(p22;q14),der(2)t(2;6;9)(p23;?;?),del(5)(q12q34),del(6)(q21),-9,-12,-13[12]/46,XX[17]	Petrella et al. [12]
17	45,X,-Y[2]/46,XY[36]	Petrella et al. [12]
18	44,XY,t(1;10)(p36.1;p13),del(3)(p21),-9,-13[11]/46,XY[9]	Alayed et al. [6]
19	46,XY,add(2)(q37),der(2)t(2;3)(q21;q27),der(3)t(3;8)(p25;q24)t(2;3),del(16)(p11.1),add(19)(q13.3)[4]/47,XY,add(2)(q37),der(2)t(2;3)(q21;q27),der(3)t(3;8)(p25;q24)t(2;3),del(16)(p11.1),add(17)(p13),+mar[2]/47,XY,add(2)(q37),der(2)t(2;3)(q21;q27),der(3)t(3;8)(p25;q24)t(2;3),del(6)(q13q21),+11,del(16)(p11.1),add(19)(q13.3)[cp3]/46,XY[10]	Alayed et al. [6]
20	47 ~ 49,XY,+8,+8,del(13)(q12q14),+21[cp4]/46,XY[7]	Alayed et al. [6]
21	44 ~ 47,XX,del(3)(p21),del(6)(q13q23),+1 ~ 6mar[cp5]/46,XX[17]	Alayed et al. [6]
22	46,XY,t(1:9)(p36.1;q34)[7]	Alayed et al. [6]
23	45,XY,del(12)(p13),-13[7]	Alayed et al. [6]
24	49,XY,+der(?)t(Y;?)(q12;?),+21,+mar[17]/46,XY[3]	Bayerl et al. [13]
25	45,XY,t(2;5)(p23;q35),–9,t(12;17)(p11;p11)[9]/44,XY,idem,–13,add(15)(p11) [13]	Bayerl et al. [13]
26	46,XY,del(6)(q23),del(17)(q21)[23]/46,XY[3]	Bayerl et al. [13]
27	37 ~ 38,XX,add(3)(p25),del(6)(q21q25),–7,add (7)(p22),–8,i(8)(q10),–9,–10,add (11)(p15),i(11)(q10),–12,–13,–15,-17,add(19)(p13)[cp12]/46,XX[3]	Rakozy et al. [14]
28	42 ~ 45,XY,del(5)(q11.2q33),add(12)(p11.2),-13,add(14)(q32),-15,+mar[cp9]/46,XY[11]	Zhang et al. [15]
29	46,XY,del(5)(q13q33)[2]/46-55,XY,+5,+5,del(5)(q13q33)*x*2,–6,+7,+8,+8,der(8)t(6;8)(p11.2;q22)*x*2,–15,+16,+17,+18,+21,+21[cp5]	Wilson and Medeiros [16]
30	46,XY,del(6)(q21q25)[12]/46,XY,del(12)(p11.2p12)[4]/46,XY,add(11)(q23)[5]	Wilson and Medeiros [16]
31	45,X,-Y[17]	Patel et al. [17]
32	47,XX,t(7;9)(p15;p24),+8	Goren Sahin et al. [18]
33	44,XY,del(5)(q13),-13,der(13)t(11;13)(q12;q32),-15	Brody et al. [2]
34	45,XY,del(7)(p12),del(9)(q12q22),-10,del(11)(q21)	Anargyrou et al. [19]
35	45,XY,der(9)t(1;9)(p22;p13),del(11)(q21),−15[10]/46XY[4]	Karube et al. [20]
36	69,XXX,t(1;6)(q21;q23),der(6)t(1;6),add(7)(p11) × 2,−9,+12,add(12)(p11) × 2,−15,−15,−15,−16,+20,−22,+1 ~ 4mar[3]/46XX[17]	Karube et al. [20]
37	44,XX,t(1;6)(q31;q25)[20]	Rossi et al. [21]
38	45,XY,-1,-3,add(6)(q?),+mar[20]	Rossi et al. [21]
39	45,XX,-13/45,XX,-22/46,XX	Eguaras et al. [22]
40	46,XY,add(9)(p24),del(11)(q22)	Chang et al. [23]
41	47,X,-Y,t(6;8)(p21;q24),+add(7)(p11.2),+der(8)t(6;8),+20[17]/46,XY (LN cells) and 48,X,-Y,t(6;8)(p21;q24),+add (7)(p11.2),+der(8)t(6;8),+20[5]/49,idem,+mar[2]/49,idem,der(8)t(6;8),?t(9;15)(p22;q15),+mar[2]/46,XY[3] (BM cells)	Nakamura et al. [24]
42	46,XY,del(12)(p12),del(17)(p11)[17]/46,XY[13]	Agapidou et al. [25]
43	44,XY,der(1)t(1;1)(q42;q11),t(7;12)(p13;p13),-13,-17,der(19)t(17;19)(q21;p13)[8]/46,XY[11]	DiGiuseppe et al. [26]
44	45,XY,der(7)t(1;7)(q11;p22),der(12;15)(q10;q10),add(13)(q12)[7]/44,idem,-9[4]/44,idem,del(3)(p25),-9[3]/46,XY[6]	Kameoka et al. [27]
45	45,XY,der(3;7)(q10;q10),t(6;19)(p21.1;p13.3),t(8;18)(q24.1;q21.1) [16]/46,XY[4]	Yu et al. [28]
46	46,XY,der(12)t(12;22)(p13;q12)del(22)(q12q12)inv(12)(p13q24.1),der(22)t(12;22)del(12)(p13p13)[10]/46,XY[10]	this study

^a^In some cases, current description of abnormal karyotypes may be slightly different from their previous literature reports. A minor modification has been made in order to follow the ISCN 2013 nomenclature guidelines as well as to integrate all findings by other means than conventional analysis (e.g., FISH and SKY) into the description of an abnormal karyotype

Thirty-four of 46 (74 %) cases of BPDCN reported had a complex karyotype (at least 3 chromosomal aberrations including at least one structural aberration), indicating that multiple recurrent chromosomal abnormalities are very common. The frequency of involvement of each chromosome is listed in Table 2. Of interest, our literature review identified the same six major chromosomal aberrancies reported by Leroux et al. [4], but with a deviant frequency for each chromosome as follows: 6 (20/46, 43 %), 12 (20/46, 43 %), 13 (20/46, 43 %), 9 (17/46, 37 %), 15 (17/46, 37 %), and 5 (15/46, 33 %). Whereas numerical aberrations were detected frequently for chromosomes 13 (18/20, 90 %), 9 (12/17, 71 %) and 15 (9/17, 53 %), we observed structural aberrations more commonly in chromosomes 6 (20/20, 100 %), 5 (14/15, 93 %) and 12 (16/20, 80 %). Further analysis examining the breakpoints and consequences of the above mentioned structural aberrations has revealed that 5q-, 6q- and/or 12p- were common with a frequency of 93 % for 5q-, 90 % for 6q-, and 88 % for 12p-. This phenomenon has been observed by Lucioni et al. [29] in a study of 21 BPDCN cases by using an array-based comparative genomic hybridization (aCGH) assay as well as by other research groups [5, 30–34]. We have an ongoing project studying the correlation between the complexity of the karyotype and outcome of this disease in patients who had been seen and followed-up in our institute.

**Table 2:** Distribution of numerical and structural chromosomal abnormalities in BPDCN

Chr.	Total (%)^a^	Numerical aberrations	Structural aberrations
X	1 (2 %)	(*n* = 0)	del(X)(q24) (*n* = 1)
Y	6 (13 %)	loss (*n* = 5)	der(?)t(Y;?); der(1)t(Y;1)(q12;q?21); t(Y;14)(q11;p11) (*n* = 3)
1	12 (26 %)	monosomy (*n* = 1)	del(1)(p22); del(1)(q42q44); der(1)t(Y;1)(q12;q?21); der(1)t(1;1)(q42;q11); t(1;6)(p21;p36.3)t(1;6)(q21;q23); t(1;6)(q31;q25); t(1;8)(q?;q?); der(1)t(1;8)(?q43;?); t(1:9)(p36.1;q34); t(1;10)(p36.1;p13); t(1;12)(?;p11); t(1;12)(q22;p13); der(1)t(1;15)(p22;q14) (*n* = 13)
2	7 (15 %)	(*n* = 0)	del(2)(p2?1); add(2)(q37); add(2)(q3?); dup(2)(?q35q37); der(2)(?); der(2)t(2;3)(q21;q27); t(2;5)(p23;q35); der(2)t(2;5)(p?;?); der(2)t(2;6;9)(p23;?;?) (*n* = 7)
3	11 (24 %)	monosomy (*n* = 1)	del(3)(p21); add(3)(p25); der(3)t(1;3)(p11;q?); t(3;5)(q?21;q?31); t(3;6)(p25;q2?3); der(3;7)(q10;q10); der(3)t(3;8)(p25;q24)t(2;3) (*n* = 11)
4	1 (2 %)		del(4)(q23) (*n* = 1)
5	15 (33 %)	monosomy (*n* = 2); trisomy (*n* = 2)	del(5)(p13); del(5)(q11.2q33); del(5)(q12q34); del(5)(q13q33); del(5)(q13q34); del(5)(q13); del(5)(q14); del(5)(q14q23); del(5)(?q33q35); add(5)(q35); t(2;5)(p23;q35); t(3;5)(q?21;q?31); der(5)t(5;11)(p10;?); der(5)t(5;13)(q21;q?) (*n* = 14)
6	20 (43 %)	monosomy (*n* = 1); trisomy (*n* = 2)	add(6)(p11.2); add(6)(q?); add(6)(q10); del(6)(q13q21); del(6)(q13q23); del(6)(q16); del(6)(q21); del(6)(q21q25); del(6)(q23); der(6)t(1;6)(q22;1q?); add(6)(q?22); t(1;6)(q21;q23); t(1;6)(p21;p36.3)t(1;6)(q31;q25); der(6)t(2;6); t(3;6)(p25;q2?3); t(6;8)(p21;q24); t(6;18)(q2?2;q22); t(6;19)(p21.1;p13.3) (*n* = 20)
7	11 (24 %)	monosomy (*n* = 1); trisomy (*n* = 1)	add(7)(p11); add (7)(p22); der(7)t(1;7)(q11;p22); der(7)del(p11.2)del(7)(q11.2q31); der(3;7)(q10;q10); t(7;9)(p15;p24); der(7)t(7;12)(?p11.2;q11); t(7;12)(p13;p13); der(7)t(7;12)(q22;q13); der(7)t(7;19)(p21;q10) (*n* = 10)
8	11 (24 %)	monosomy (*n* = 1); trisomy/tetrasomy (*n* = 3)	i(8)(q10); add(8)(q2?4); t(6;8)(p21;q24); der(8)t(6;8)(p21;q24); t(8;14)(q24.1;q32); t(8;18)(q24.1;q21.1) (*n* = 9)
9	17 (37 %)	monosomy (*n* = 12)	add(9)(p24); del(9)(q12q22); der(9)t(1;9)(p22;p13); t(1:9)(p36.1;q34); t(7;9)(p15;p24); t(9;15)(p22;q15) (*n* = 7)
10	3 (7 %)	monosomy (*n* = 2)	t(1;10)(p36.1;p13) (*n* = 1)
11	10 (22 %)	monosomy (*n* = 1); trisomy (*n* = 1)	add (11)(p15); i(11)(q10); del(11)(q14q23); del(11)(q21); del(11)(q22); del(11)(q23); add(11)(q23); inv(11)(p11q21); del(11)(q13q23) (*n* = 9)
12	20 (43 %)	monosomy (*n* = 3)	add(12)(p11); add(12)(p11.2); der(12)(?); del(12)(p11.2p12); del(12)(p12); del(12)(p12p13); del(12)(p13); der(12)t(1;12)(?;p11); der(12)t(1;12)(q22;p13); der(12)t(5;12)(?;p11); t(7;12)(p13;p13); t(12;17)(p11;p11); r(12); der(12)t(12;22)(p13;q12)del(22)(q12q12)inv(12)(p13q24.1) (*n* = 16)
13	20 (43 %)	monosomy (*n* = 18)	add(13)(q12); del(13)(q12q22); del(13)(q12q14); del(13)(q1?3q2?1); der(13)t(11;13)(q12;q32); der(13)t(13;21)(q10;q10) (*n* = 4)
14	3 (7 %)	monosomy (*n* = 1)	add(14)(q32); der(14)t(Y;14)(q11;p11) (*n* = 2)
15	17 (37 %)	monosomy (*n* = 9)	add(15)(p11); inv(15)(q1?4q2?3); der(15)t(1;15)(p?;q21); ?t(9;15)(p22;q15); der(12;15)(q10;q10); der(15;18)(q10;q10); r(15) (*n* = 8)
16	4 (9 %)	monosomy (*n* = 1); trisomy (*n* = 1)	del(16)(p11.1); t(16;16)(q?;q?) (*n* = 2)
17	9 (20 %)	monosomy (*n* = 3); trisomy (*n* = 1)	add(17)(p11); del(17)(p11); add(17)(p13); del(17)(q21); t(12;17)(p11;p11) (*n* = 6)
18	6 (13 %)	monosomy (*n* = 2); trisomy (*n* = 1)	add(18)(q11.2); t(6;18)(q2?2;q22); der(15;18)(q10;q10) (*n* = 3)
19	7 (15 %)	monosomy (*n* = 2)	add(19)(p13); add(19)(p13.3); der(19)t(1;19)(q23;p13); der(19)t(3;19)(p21;q13); t(6;19)(p21.1;p13.3); t(7;19)(p21;q10); der(19)t(17;19)(q21;p13) (*n* = 6)
20	4 (9 %)	trisomy (*n* = 3)	der(20;21)(?p11;?q22) (*n* = 1)
21	6 (13 %)	monosomy (*n* = 1); trisomy (*n* = 4)	der(20;21)(?p11;?q22); der(21;21)(q21;q11)ins(21;12)(q11;?) (*n* = 1)
22	5 (11 %)	monosomy (*n* = 3)	der(22)t(12;22)del(12)(p13p13); i(22)(q10) (*n* = 2)
M	11 (24 %)	(*n* = 0)	(*n* = 11)
R	4 (9)	(*n* = 0)	r(12); r(13); r(15); r(?) (*n* = 4)

^a^The majority of cases had a complex karyotype, containing multiple numerical and structural aberrations. M: marker chromosome; R: ring chromosome

The 12p- is considered to be one of the most common structural aberrations in BPDCN. Two major target genes/loci located on the 12p have been identified as potential hotspots in the literature, *CDKN1B* and *ETV6*. The *CDKN1B* gene is located on 12p13.1p12 spanning from 12,717,270 to 12,722,383 (5114 bp, GRCh.38.p2). The protein encoded by this gene, p27 (also known as KIP1), is a kinase inhibitor and an atypical tumor suppressor through regulation of the activity of cyclin-dependent kinases (Cdks). Therefore, dysfunction of *CDKN1B* plays a role in pathogenesis and metastasis of multiple cancers, such as breast cancer, prostate cancer and leukemia [39]. Loss of the *CDKN1B* locus has been reported in over 60 % of BPDCN cases, including cases with a normal karyotype [5, 29–32]. Due to the size of this gene, its loss has been detected only by array comparative genomic hybridization assay in the literature. Immunohistochemistry studies have further confirmed weak expression of p27 protein in almost all these cases. *ETV6*, also known as *TEL*, *THC5* or *TEL/ABL* gene, is located on 12p13 spanning from 11,649,854 to 11,895,402 (245,549 bp), less than 1 Mb apart from *CDKN1B*. Both genes were simultaneously lost in the cases mentioned above. *ETV6* is a gene with the characteristics of a tumor suppressor gene and encodes an ETS family transcription factor. *ETV6* has been shown to play an important role in the pathogenesis of various types of leukemia, mostly through forming fusion genes with over 40 different translocation partners [40, 41]. In BPDCN (Tables 1 and 2), over 40 % of cases had a structural abnormality involving 12p, very likely resulting in a 12p- at various breakpoints and including a deletion of *CDKN1B* and *ETV6* in most cases by roughly analyzing the breakpoints. Some of these cases might have had an *ETV6* rearrangement as well. *ETV6* (approximately 250 Kb of size) is a good target for FISH testing and other studies have demonstrated a deletion or rearrangement of *ETV6* by FISH testing in BPDCN cases with or without an obvious 12p-; the deletion can be monoallelic or biallelic [5, 29]. Therefore, 12p-, more specifically a deletion or rearrangement of *CDKN1B* and/or *ETV6*, may deserve further investigations as potential markers of BPDCN.

Chromosome 22 abnormalities appear to be rare in BPDCN (5/46, 11 %) (Tables 1 and 2). Three cases reported so far had a numerical change and 2 other cases (including the one in this study) had a structural aberration of chromosome 22. However, chromosomal aberrations involving a chromosome 22 might have been underestimated, most likely due to technical limitations including: size of chromosome 22 (one of the smallest chromosomes with limited banding patterns); extremely low karyotyping resolution in cancer cases; and high percentage of complex karyotypes and marker chromosomes in BPDCN cases. In our case, a *EWSR1* rearrangement with a deletion of the *5′EWSR1* was detected. *EWSR1* rearrangement has been reported mostly in soft tissue tumors [42–44], also rarely in hematologic malignancies [37, 45, 46], but only in one BPDCN case previously [37]. The *EWSR1* gene has a large number of fusion partners, including members of the ets family, such as *FLI1*, *ERG* and *ETV1* but not *ETV6* [42–44]. As mentioned above, according to the locations of the remaining *5′ETV6* and *3′EWSR1*, an *ETV6/EWSR1* fusion can be excluded in this case. However, due to occurrence of inversion in the affected chromosome 12 and partial deletion of both *3′ETV6* and *5′EWSR1*, gene fusions between *ETV6* and a partner gene on 22q as well as between *EWSR1* and a partner gene on 12q cannot be completely excluded. The biological implications of these *EWSR1* aberrations are unknown.

Clinical manifestation of BPDCN vary among patients. Most BPDCN patients present with one or more skin lesions with or without BM or LN involvement. The patient in this study presented with progressive LN enlargement as the only notable finding. His BM tested by multiple means was negative for BPDCN involvement. However, the same chromosomal aberrations were detected in his LN with BPDCN as well as BM without BPDCN. Combined morphologic and FISH analysis further confirmed that the BM cells carrying the chromosomal aberrations were morphologically normal. One explanation is that the chromosomal abnormalities in our case may constitute an initiating event within a hematopoietic stem cell precursor and that a second hit in a cell capable of acquiring phenotypic features of plasmacytoid dendritic cells is required for BPDCN to develop (Fig. 3). This hypothesis is intriguing particularly since a link between BPDCN and myeloid malignancies has long been observed, even though the pathogenic link between these entities remains unknown. Indeed, many patients with BPDCN have shown to develop acute myeloid leukemia (AML) [3, 6, 28]. In addition, a sizeable subset of BPDCN involving the BM has been reported to be associated with myelodysplastic features at the morphologic and/or cytogenetic levels [6]. Another explanation is that a minimal BM dissemination of BPDCN and growth advantage of neoplastic cells in ex vivo culture may contribute to the results in our case.

In summary, over 50 % of BPDCN cases have chromosomal abnormalities, with more than 70 % of BPDCN cases exhibiting a complex karyotype. Monosomy 13/13q-, 12p-, 6q-, monosomy 15/15q-, 5q- and monosomy 9 are characteristic chromosomal abnormalities in BPDCN. The 12p- is one of the most common structural aberrations in BPDCN, and a deletion/rearrangement of *CDKN1B* and/or *ETV6* on 12p is often detected. These two genes, together with *EWSR1* on 22q, may deserve further investigations as potential BPDCN markers.

## Conclusion

This is the first case of BPDCN that carried a translocation between chromosomes 12 and 22, followed by a subsequent pericentric inversion of the abnormal chromosome 12, and that resulted in a simultaneous partial deletion of *3′ETV6* and *5′EWSR1*. Analyzing all 45 BPDCN cases with abnormal karyotypes available in the literature plus this case, 6 major chromosomal targets are identified in BPDCN: chromosomes 6 (20/46, 43 %), 12 (20/46, 43 %), 13 (20/46, 43 %), 9 (17/46, 37 %), 15 (17/46, 37 %), and 5 (15/46, 33 %). Deletion of 12p (12p-) is one of the most common structural aberrations, and the *ETV6* and *CDKN1B* on 12p deserve further investigations as potential markers of BPDCN.

## Consent

Written informed consent was obtained from the patient for publication of this Case report and any accompanying images. A copy of the written consent is available for review by the Editor-in-Chief of this journal.

## Abbreviations

aCGH: array-based comparative genomic hybridization
BM: bone marrow
BPDCN: blastic plasmacytoid dendritic cell neoplasm
Cdks: cyclin-dependent kinases
FISH: fluorescence in situ hybridization
LN: lymph nodes
PB: peripheral blood
SKY: spectral karyotyping
WCP: whole chromosome painting
